# Quantitative Infection Dynamics of Cafeteria Roenbergensis Virus

**DOI:** 10.3390/v10090468

**Published:** 2018-08-31

**Authors:** Bradford P. Taylor, Joshua S. Weitz, Corina P. D. Brussaard, Matthias G. Fischer

**Affiliations:** 1 Program for Computational Biology, Memorial Sloan Kettering Cancer Center, New York, NY 10065, USA; taylorb2@mskcc.org; 2 School of Biological Sciences and School of Physics, Georgia Institute of Technology, Atlanta, GA 30332, USA; jsweitz@gatech.edu; 3 Department of Marine Microbiology and Biogeochemistry, NIOZ Royal Netherlands Institute of Sea Research, and University of Utrecht, P.O. Box 59, 1790 AB Den Burg, Texel, The Netherlands; Corina.Brussaard@nioz.nl; 4 Aquatic Microbiology, Institute for Biodiversity and Ecosystem Dynamics, University of Amsterdam, P.O. Box 94248, 1090 GE Amsterdam, The Netherlands; 5 Department of Biomolecular Mechanisms, Max Planck Institute for Medical Research, 69120 Heidelberg, Germany

**Keywords:** giant viruses, multiple infections, virus factories, infection modeling, CroV

## Abstract

The discovery of giant viruses in unicellular eukaryotic hosts has raised new questions on the nature of viral life. Although many steps in the infection cycle of giant viruses have been identified, the quantitative life history traits associated with giant virus infection remain unknown or poorly constrained. In this study, we provide the first estimates of quantitative infection traits of a giant virus by tracking the infection dynamics of the bacterivorous protist *Cafeteria roenbergensis* and its lytic virus CroV. Leveraging mathematical models of infection, we quantitatively estimate the adsorption rate, onset of DNA replication, latency time, and burst size from time-series data. Additionally, by modulating the initial ratio of viruses to hosts, we also provide evidence of a potential MOI-dependence on adsorption and burst size. Our work provides a baseline characterization of giant virus infection dynamics relevant to ongoing efforts to understand the ecological role of giant viruses.

## 1. Introduction

Numerous eukaryotic viruses larger than 150 nm in diameter and 200 kbp in genome size that infect marine microbes have been identified in recent years [1,2,3,4]. These “giant” viruses are hypothesized to have substantial ecological impacts on hosts including inducing population collapses [5] and controlling algal blooms [6,7]. Virus-induced lysis can also cause major changes to food webs by diverting the flow of nutrients towards lower trophic levels in a process termed the “viral shunt” [8,9], to which giant viruses are likely to contribute [3,10]. Yet, quantitative infection parameters that influence giant virus dynamics remain largely unknown. A key challenge in estimating giant virus life history traits is that several conventional methods in virology such as the plaque assay are not available, or can be applied only to certain hosts [11,12].

The giant Cafeteria roenbergensis virus (CroV) is a parasite of the bacterivorous protist *Cafeteria roenbergensis*, a heterotrophic nanoflagellate that is widespread in marine environments [13,14]. Like many other giant viruses (and unlike bacteriophages), CroV reproduces in localized cytoplasmic structures of viral origin termed virion factories [15,16,17]. Following transcription and translation of viral genes, new virus particles self-assemble at the factory periphery. Of note is that the number of virion factories per cell for the giant mimivirus has been observed to increase with increasing multiplicity of infection (MOI), i.e., with increasing ratio of virus particles to host cells [18]. Observations of virion factories merging during infection suggest that infection dynamics may be complicated by multiple infection events [18]. Hence, it is not yet known whether life history traits associated with giant virus infections vary as a function of MOI (see [7,19]).

To estimate life history traits and identify potential MOI dependencies, we tracked the infection dynamics of CroV across a 24-h period with near-hourly sampling. Overall, we quantitatively estimate the growth rate of the protist host and CroV life history traits including adsorption rate, onset of DNA replication, latent period, and burst size. As we detail, our time-series framework enables us to estimate quantitative infection parameters and provides evidence for potential declines in the efficiency and rate of virus spread with increasing MOI.

## 2. Materials and Methods

Suspension cultures of the flagellate *C. roenbergensis*, strain RCC970-E3 (a clonal derivative of strain RCC4623 of the Roscoff Culture Collection), were grown in f/2 artificial seawater medium supplemented with 0.05% (*w*/*v*) Bacto${}^{\mathrm{TM}}$ yeast extract (Becton, Dickinson and Company, Heidelberg, Germany) to stimulate growth of the mixed bacterial community that serves as the food source for *C. roenbergensis*, as described previously [20]. Exponentially growing cells were diluted with medium to a density of 7.0 × 10${}^{5}$ cells per mL and nine 30 mL aliquots (containing 2.1 × 10${}^{7}$ cells each) were dispensed in 125 mL polycarbonate Erlenmeyer flasks. Three aliquots each served as biological triplicates for the uninfected control cultures, CroV-infected MOI = 1 cultures, and CroV-infected MOI = 10 cultures. Cultures were infected with a suspension of Cafeteria roenbergensis virus (CroV) strain BV-PW1 [14] at an infectious titer of 1.0 × 10${}^{8}$ CCID50/mL (the cell culture infectious dose at which 50% of the cultures lyse). Virus titers were determined by end-point dilution assays as described previously [20].

At t = 0 h post infection (hpi), 210 μL of CroV suspension were added to each of the three MOI = 1 cultures, 2.1 mL of CroV suspension were added to each of the three MOI = 10 cultures, and 2.1 mL of sterile medium were added to uninfected cultures as a control. The cultures were then incubated for 15 min at 22 °C with gentle agitation. In order to remove any free CroV particles, the cultures were transferred to 50 mL polycarbonate tubes and centrifuged for 10 min at 4500 rcf, 20 °C in an Eppendorf 5804R centrifuge. The supernatants were decanted, the cell pellets were resuspended in 30 mL f/2 medium, and the centrifugation procedure was repeated two more times. After the final wash step, the cells were resuspended in 30 mL f/2 medium with 0.05% (*w*/*v*) yeast extract, transferred to 125 mL polycarbonate Erlenmeyer flasks, and incubated at 22 °C with 50 rpm shaking.

Aliquots for qPCR, FCM, and microscopy analyses were taken from each culture every hour, starting immediately after infection (0 hpi). To cover the entire infection cycle of CroV, the experiment was conducted twice, once to sample from 0 to 9 hpi as well as the 12 and 24 hpi time points, and once to sample from 12 to 24 hpi. Throughout the manuscript, we designate these sampling times as “early” and “late”, respectively. Cell concentrations were measured by staining a 10 μL aliquot of the suspension culture with 1 μL of Lugol’s Acid Iodine solution and counting the cells on a hemocytometer (Neubauer Improved Counting Chamber, VWR, Darmstadt, Germany). Samples (200 μL) for DNA extraction and subsequent qPCR analysis were processed as described previously [20]. A 128 bp long fragment of the *crov283* gene for the VV D11-like transcription factor (GenBank Accession No: ADO67316.1) was amplified by primers CroV-qPCR-9 and CroV-qPCR-10 and used as an approximation for CroV genome copies. PCR conditions have been described previously [20]. For FCM analysis, two 490 μL aliquots (one for FCM analysis, one for backup) were taken per time point and mixed with 10 μL 25% glutaraldehyde in 2 mL cyrovials. After a 20 min incubation at 4 °C, the fixed samples were frozen in N${}_{2}$(l) and stored at −80 °C until further analysis. Flow cytometry of viral particles was carried out as described previously [21].

We analyzed the subsequent data by leveraging nonlinear model fitting and statistical analyses to estimate infection dynamics parameters. The statistical analyses were performed over the three biological replicates for host growth (with technical duplicates), viral qPCR (with technical duplicates), and viral FCM. Models are introduced in the relevant sections and statistical analyses are specified for each life history trait. We performed nonlinear model fitting by log-transforming data points and utilizing standard least-square minimization techniques. Additionally, we developed a series of paired *t*-tests to compare samples between adjacent time points in order to identify life history traits related to timing. Note, we used paired *t*-tests in lieu of conventional change-point analyses that require finer grained sampling [22].

## 3. Results

### 3.1. Host Growth Rate in the Absence of CroV

**Figure 1:**
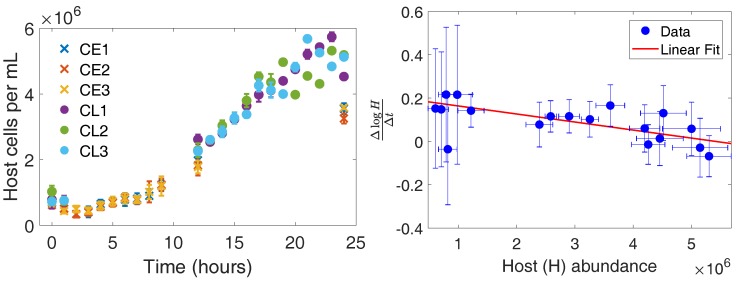
*Cafeteria roenbergensis* growth in the absence of CroV. (**Left**) Cell densities over time. The 24-h experiments were split into two 12-h periods of intense sampling for feasibility. Each color refers to a different set of replicates where CL refers to control–late and CE refers to control–early. The numbers refer to biological replicates. Samples for the “early” experiment occurred at {0,1,2,3,4,5,6,7,8,9,12,24} h and for the “late” experiment occurred at {0,1,12,13,14,15,16,17,18,19,20,21,22,23,24} h. (**Right**) Fitting a logistic growth model to host growth between sampled points. We average the abundances at each time point to reduce noise when fitting the model.

**Figure 2:**
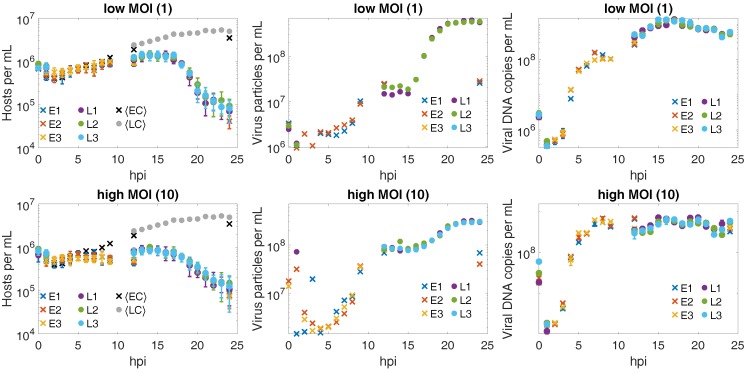
Infection dynamics at low and high MOI. (**Top Row**) For the low MOI case, (left) host densities are estimated from microscopy counting, (middle) external virus particles are estimated from flow cytometry, and (right) total viral DNA copies are estimated from qPCR analysis. The same scheme applies to the high MOI experiments (**Bottom Row**). Samples for the “early” (E) experiment were taken at {0,1,2,3,4,5,6,7,8,9,12,24} h post infection and “late” (L) samples were taken at {0,1,12,13,14,15,16,17,18,19,20,21,22,23,24} h post infection. The numbers refer to replicates. Average host population densities are shown in black for the early control (EC) experiments and in gray for the late control (LC) experiments. Error bars capture variation between technical replicates. There are no error bars for the FCM data because measurements were only performed once for each sample.

Host growth in the absence of CroV acts as a control to establish host dynamics in batch culture. Figure 1 shows the host abundance over time in the absence of CroV. The hosts maintain growth and appear to reach a carrying capacity within 24 h. The initial reduction of hosts between 0 and 1 h is likely due to the repeated centrifugation and removal of supernatant. Further loss and lack of growth at early times is likely a bottling effect as it was also observed in the infected cultures (Figure 2). Possible explanations include stress-induced apoptosis and slow growth due to a lag-phase after addition of fresh medium. These phenomena have been observed for protist systems, but not explicitly for *C. roenbergensis*. Hence, we merely note the observation with no claims for the mechanism.

We estimate the infection-free host doubling time and batch carrying capacity by fitting the growth dynamics to a logistic growth differential equation model $\frac{dH}{dt}=rH\left(1-\frac{H}{K}\right)$, where *H* is the host abundance, *r* is the host growth rate at low densities, and *K* is the carrying capacity of the batch system. Fitting logistic growth to the full dynamics is problematic since the initial host densities may differ between our early and late experiments. Instead, we utilize a numerical approximation of dlog(H)/dt given τ, the time between adjacent samples:

(1) $$\begin{array}{ccc} \frac{d\log(H)}{dt}\approx \frac{\log(H(t+\tau ))-\log(H(t))}{\tau } & = & r\left(1-\frac{H}{K}\right) \end{array}$$

The right side of Figure 1 shows our regression to growth dynamics in which we estimate r=0.200±0.075 h${}^{-1}$ and $K=5.41\times 10^{6}\pm 3.84\times 10^{6}$ hosts. Note, our errors here and throughout the rest of the paper refer to standard deviations after accounting for propagation of uncertainty [23]. The estimated host doubling time is $T_{d}=\frac{\log\left(2\right)}{r}=3.45\pm 1.29$ h, which is consistent with the previous reported range of 3.3–8.3 h [24].

### 3.2. CroV Infection Parameters

In the CroV-infected cultures, we measured host and viral abundances at near-hourly frequency for 24 h following infection (Figure 2). Overall, the population dynamics plotted in log-scale qualitatively show two periods of lysis featuring virus particle production and reduced host growth.

The adsorption rate can be estimated from the reduction in the viral DNA copies between 0 and 1 hpi. Between these times, the sample underwent repeated centrifugation, removal of the supernatant, and resuspension (see Methods). This process removes the majority of free virus particles and, to a much lesser extent, some host cells which may be infected or uninfected. Thus qPCR measures the total viral titer at 0 hpi and the remaining infecting viral abundance at 1 hpi leading to:

(2) $$\begin{array}{ccc} \Delta V & = & V(t=1\;\mathrm{hpi})-V(t=0\;\mathrm{hpi})\\ & = & -\overset{\mathrm{unadsorbed}\;\mathrm{free}\;\mathrm{viruses}\;\mathrm{lost}}{\overset{︷}{V\left(t=0\;\mathrm{hpi}\right)\left(1-P_{\mathrm{infect}}\right)}}-\overset{\mathrm{viruses}\;\mathrm{infecting}\;\mathrm{hosts}\;\mathrm{lost}}{\overset{︷}{V\left(t=0\;\mathrm{hpi}\right)P_{\mathrm{infect}}\bar{f_{H}}}} \end{array}$$

where *V* refers to viral DNA copies both inside and outside the cell as measured by qPCR. The second loss term depends on the probability that a virus infects a host $P_{\mathrm{infect}}$ and the fraction of hosts lost due to washout, $\bar{f_{H}}$. We estimate $\bar{f_{H}}$ as the average decrease in host abundance between 0 and 1 hpi. Note, we assume there is negligible host growth during this period. For each replicate we can solve (2) for $P_{\mathrm{infect}}$. Biophysical models connect the adsorption rate, ϕ, to the probability of infection, $P_{\mathrm{infect}}$ [25]. Specifically, we assume a one-step viral infection process leading to $P_{\mathrm{infect}}\;=\;1-e^{-\phi \bar{H(t=0\;\mathrm{hpi})}T}$, where the inoculation time is T=0.25 h.

**Figure 3:**
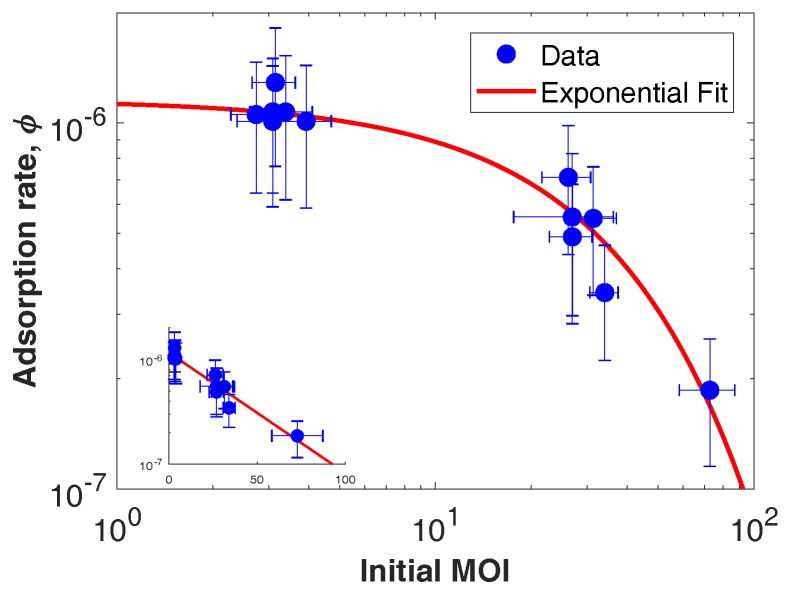
Adsorption rate as a function of initial MOI. The main figure is in log-log axes, and the inset is in log-linear axes. Adsorption rate, ϕ, was estimated using (2). The initial MOI was estimated from the ratio of the viral genome copies as measured by qPCR at zero hpi to the measured host abundance at 0 hpi averaged across replicates. Averaging was done to minimize error due to manual counting of hosts.

Solving (2) reveals that the adsorption rate decreases approximately exponentially with increasing MOI (Figure 3, fit in red). The exponential fit ($R^{2}=0.94$) appears curved because we use a logarithmic *x*-axis to emphasize the difference in adsorption rates between high and low initial MOI. The mean adsorption rate is $\phi_{\mathrm{low}}=1.09\times 10^{-6}\pm 4.57\times 10^{-7}\;\frac{\mathrm{mL}}{h}$ for low MOI and $\phi_{\mathrm{high}}=4.72\times 10^{-7}\pm 2.63\times 10^{-7}\;\frac{\mathrm{mL}}{h}$ for high MOI. These inferred adsorption rates are slower than the diffusion-limited estimate $\phi =2.2\times 10^{-6}\;\frac{\mathrm{mL}}{h}$ as derived from biophysical models assuming Stokes-Einstein relationships [26].

We estimate life history traits related to timing, such as the onset of DNA replication and latent period, by comparing the viral counts between adjacent time points. Specifically, we use one-sided paired *t*-tests to identify when the change in count data is significantly positive. For transparency, we present the *p*-values for all comparisons since our low number of replicates reduces the robustness of significance testing.

**Figure 4:**
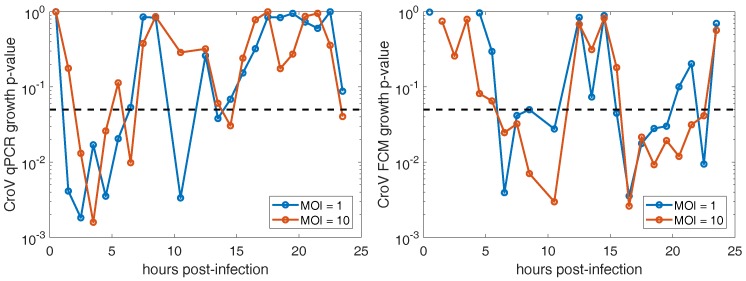
Significance values for identifying virus production. For a given datapoint at the plotted time, lower *p*-values correspond to higher probability of viral production between the current and previous time point with *p* = 0.05 noted in dotted black line. (**Left**) Viral DNA copies increase as measured by qPCR. (**Right**) Extracellular viral particle increase as measured by FCM.

The onset of CroV DNA replication, as estimated by the first statistically significant increase in viral DNA copies measured by qPCR occurs at $\tau_{e}=1.5\pm 1$ hpi for MOI = 1 and $\tau_{e}=2.5\pm 1$ hpi for MOI = 10 (see Table 1). The *p*-values of the temporal paired *t*-tests for qPCR data are shown on the left side of Figure 4 where we use p=0.05 as the threshold for significance. However, we caution over-interpreting changes in significance between adjacent pairs of points due to our small number of replicates.

The latent period, as estimated by the first statistically significant increases in extracellular viral particles measured by FCM lasts for $\tau_{L}=5.5\pm 1$ h and this initial period of particle release continues for several hours (see Table 1). The *p*-values of the temporal paired *t*-tests for FCM data are shown on the right side of Figure 4. The latent period extends beyond 9 hpi since the *p*-value at 12 hpi reflects the change in viral abundance between 9–12 hpi. A second period of virus particle release begins following 15 hpi and sustains through 22 hpi. During this period, visible host lysis coincides with the release of the majority of CroV particles (see Figure 2).

We estimate the burst size, β, during periods of lysis from the changes in the population sizes $\beta \approx -\frac{\Delta V}{\Delta H}$ as measured via host counts and FCM. We estimate only the burst size from the second period of lysis because host and viral population changes during the first period of lysis are non-negligibly affected by host-growth and secondary viral infection. We calculate the population changes between the time point immediately preceding lysis (15 hpi) and the final point (24 hpi), e.g., ΔH=H(t=24hpi)−H(t=15hpi) yielding β=[461±56,477±125] in the low MOI case and β=[315±58,314±87] in the high MOI case. Note, the two values represent estimates based on FCM measurements on two of the three replicates and the associated error results from technical variation in estimating host abundances. The average MOI for the second period of lysis is not available since this depends on the infection dynamics following the first period of lysis. However, we expect fewer hosts initially infected in the low MOI case and, in turn, a larger MOI in the high MOI case for the second period of lysis. Together, this gives indirect evidence that burst size decreases with increasing MOI.

### 3.3. Integration of Life History Traits into a Mechanistic Model of CroV-Host Population Dynamics

In the prior subsections, we estimated CroV life history traits relevant to infection of *C. roenbergensis*. A summary of our results is presented in Table 1.

**Figure 5:**
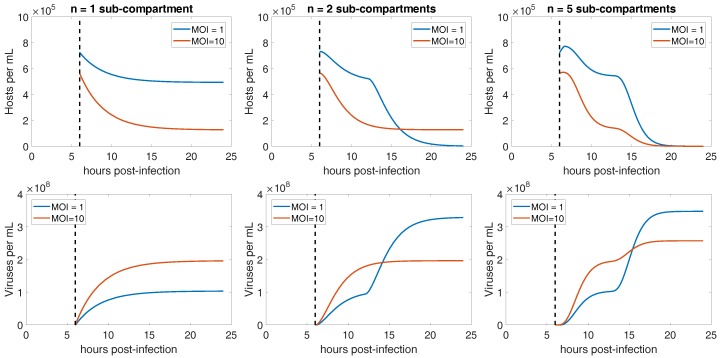
Simulated infection dynamics across different variation in latent period. (**Top Row**) Host dynamics for low and high MOI with (left) high variation in latent periods obtained using a single sub-compartment of infection, *n* = 1, (middle) moderate variation in latent periods obtained using two sub-compartments of infection, *n* = 2, and (right) low variation in latent periods obtained using five sub-compartments of infection, *n* = 5. (**Bottom Row**) Virus dynamics for low and high MOI with (left) high variation in latent periods obtained using a single sub-compartment of infection, *n* = 1, (middle) moderate variation in latent periods obtained using two sub-compartments of infection, *n* = 2, and (right) low variation in latent periods obtained using five sub-compartments of infection, *n* = 5.

One goal of estimating life history traits is to inform dynamical models of giant virus reproduction. Here we apply the empirically derived parameters to a model matching our experimental set-up, including infection sub-compartments [27]:

(3) $$\begin{array}{ccc} \frac{dH}{dt} & = & rH\left(1-\frac{H}{K}\right)-\phi HV\\ \frac{dI_{0}}{dt} & = & \phi HV-\phi H\left(t-\tau_{e}\right)V\left(t-\tau_{e}\right)\\ \frac{dI_{1}}{dt} & = & I_{0}\left(t-\tau_{e}\right)-\lambda I_{1}\\ \vdots \\ \frac{dI_{n}}{dt} & = & \lambda I_{n-1}-\lambda I_{n}\\ \frac{dV}{dt} & = & \beta \lambda I_{n}-\phi HV-mV \end{array}$$

where *H* denotes uninfected hosts, $I_{0}$ denotes infected hosts unable to lyse, $I_{n}$ denotes infected hosts in the *n*th sub-compartment of infection, and *V* denotes extracellular viruses. For generality we include viral decay, *m*; however, we set m=0 for our simulations due to the short duration of the experiment relative to months over which CroV stocks remain infectious. The parameters are the best estimates of life history traits as listed in Table 1. The number of sub-compartments, *n*, is unknown and controls the variance of the latent period by appropriately scaling the transition rate, λ=nΛ where 1/Λ is the average time spent as an infected cell after the onset of DNA replication. Simulations of the model in Figure 5 show that the periods of lysis become more distinguishable with less variation in the total latent period, i.e., more sub-compartments of infection, *n*.

## 4. Discussion

By leveraging empirical data and mathematical models of infection, we estimated several life history traits related to infection by the giant DNA virus CroV (see Table 1 for summary). We tracked the population dynamics of the host by manual counting, external virus capsids by FCM, and viral gene copies by qPCR. We attempted to minimize the error of our estimates by sampling at a near-hourly frequency across a 24-h period. Additionally, we propagated the uncertainty of measurements in our calculations in order to constrain life history trait estimates from regression techniques. We compared our estimates across different regimes of MOI by performing the experiments with two different ratios of virus inoculum to host cells. Our results show that infection cycle timing is insensitive to MOI whereas the adsorption rate and burst size of viruses may both decrease with increasing MOI.

Our results represent the first in-depth characterization of the infection dynamics of a giant virus that utilizes virion factories for reproduction. Prior work on certain mimivirus strains, which focused on culturing methodology as opposed to estimating life history traits, reported higher virion production rates at lower MOIs [28]. Similarly, Brown et al. observed a reduction in production of AaV at high MOI [7]. These reports are consistent with our findings. However, it is not clear whether AaV forms virion factories [19]. The use of near-hourly sampling helps explain why the MOI = 1 infections resulted in roughly double the number of virus particles after 24 hpi compared to the MOI = 10 infections. Specifically, fewer hosts initially infected leave more hosts to reproduce. This increases the number of hosts available for infection during a second period of lysis in the low MOI treatment. Interestingly, in contrast to the FCM data, we observe roughly the same concentration of viral gene copies by the end of the experiment in both the low and high MOI experiments. This may indicate that capsid production and virion maturation are the resource-limiting steps, rather than viral DNA replication. The generality of this result is unclear as bottlenecks in viral production may depend on host state and, in turn, the nutrient composition of the environment.

We were surprised to see the appearance of free viral particles as early as 6 hpi since a previous study in *C. roenbergensis* strain E4-10 did not observe host cell lysis before 12 hpi [14]. The timing discrepancy with previous reports may be accounted for by different growth conditions for the host, and host strain-specific differences. The present choice of optimized growth conditions facilitated a relatively short host doubling time which may account for a shorter latent period as observed in other host-virus systems [29,30,31]. Overall, the disagreement between our quantified latent period and previous observations of viral factory timing warrants further study with respect to differences in CroV life history traits in different host strains. While our population dynamics data are not in conflict with interpreting the viral production as a period of lysis, alternative explanations may be possible. For example, it is possible that our FCM measurements were sensitive to non-CroV virus-like particles. Additionally, there may be previously unobserved heterogeneity in the timing of virus factory emergence. The observed viral production dynamics dictate that alternatives should account for a period of stasis before the second observed period of viral production.

The mechanistic basis for variation in life history traits remains unknown. Previous work on viral infection has suggested that elemental constraints generally limit the burst size [32] with examples of limitation observed for nitrogen, phosphorous, and iron [33,34,35,36,37,38]. Virus factories complicate the resource constraints as multiple infecting viruses may lead to multiple, spatially independent virus factories [18]. It is not known whether virus factories interact—whether cooperatively or antagonistically—when utilizing resources during the infection process. For example, if a single virus factory determines the lysis time then the distribution of lysis times should differ between lower and higher MOI infection whereas the range of lysis times would not be affected. Moreover, if lysis timing is determined by a single virus factory, then previously observed scalings of latent periods and burst size may not apply to viruses that utilize virus factories [39]. Additional work is required to resolve the effect of virus factory dynamics on emergent life history traits.

The present study provides estimates of giant virus life history traits that constitute a baseline for comparison, whether in other hosts, for other giant viruses, or during coinfection of giant viruses with virophages. Virophages are viruses that engage in a hyper-parasitic lifestyle with giant viruses that reproduce via virus factories [40]. Like giant viruses, the ecological impact of virophages is poorly understood. Virophage coinfection can reduce the production of giant viruses [17,40,41], perhaps enabling long-term coexistence of hosts, giant viruses, and virophages in marine environments [20]. Building upon this experimental system may provide a path forward towards a broader understanding of the link between cell-level dynamics and the ecology and global impact of giant viruses, virophages, and their hosts. 

## Figures and Tables

**Table 1:** Life history traits of CroV and its host. We calculated *p*-values for parameters with multiple values across each MOI using *t*-tests.

Life History Trait	Low MOI (1)	High MOI (10)	*p*-Value
Host doubling time	3.5±1.3 h (from control)	3.5±1.3 h (from control)	-
Onset of viral DNA replication	1.5±0.5 h	2.5±0.5 h	-
Latency time	5.5±0.5−10.5±1.5 h	5.5±0.5−10.5±1.5 h	-
Adsorption rate	$1.1\times 10^{-6}\pm 4.6\times 10^{-7}\;\frac{\mathrm{mL}}{h}$	$4.7\times 10^{-7}\pm 2.6\times 10^{-7}\;\frac{\mathrm{mL}}{h}$	3.2 × 10${}^{-5}$
Burst size	470±100 virus particles	310±70 virus particles	2.5 × 10${}^{-3}$

## Abbreviations

The following abbreviations are used in this manuscript:

CroV	Cafeteria roenbergensis Virus
hpi	hours post infection
MOI	multiplicity of infection
FCM	flow cytometry
qPCR	quantitative polymerase chain reaction
